# On the way to deprescribing levothyroxine in subclinical hypothyroidism in general practice – the patients’ perspective of enablers and barriers

**DOI:** 10.1080/13814788.2025.2571605

**Published:** 2025-10-27

**Authors:** M. Rennert, A. K. Geier, L. Christ, S. Hager, S. Scheibe, A. Rettich, J. Schübel, T. Deutsch, M. Bleckwenn, K. Voigt

**Affiliations:** a Institute for General Practice, Faculty of Medicine, Leipzig University, Leipzig, Germany; b Department of General Practice, Faculty of Medicine and University Hospital Carl Gustav Carus, TUD Dresden University of Technology, Dresden, Germany

**Keywords:** Deprescribing, qualitative research, patient involvement, levothyroxine, subclinical hypothyroidism

## Abstract

**Background:**

Recent studies indicate that the synthetic thyroid hormone levothyroxine (LTX) in many cases has no beneficial effects on patients with subclinical hypothyroidism. Still, prescriptions are increasing worldwide. If there is no clear indication for treatment, patients treated with LTX should be offered a deprescribing trial according to current guidelines. However, there is currently no protocol for deprescribing LTX in primary care.

**Objectives:**

We aimed to explore patients’ enablers and barriers towards deprescribing levothyroxine in primary care to inform the further participatory development of a deprescribing strategy.

**Methods:**

Based on the COREQ checklist, focus group discussions were conducted with patients and general practitioners as well as patients only in 2024. Participants ranked the five most crucial enablers and barriers. Transcripts and prioritised elements were examined using the qualitative content analysis method according to Kuckartz.

**Results:**

Patients frequently felt misinformed about their condition and the prescription of LTX. A change in their medications raised doubts and uncertainties. However, the potential advantages and opportunities of deprescribing were compelling: a (re)gain of quality of life, a decrease in probable drug side effects, savings of time and cost. Mostly, patients welcomed a gradual and managed deprescribing under their general practitioner’s supervision.

**Conclusion:**

Patients wished for medical information to reduce their doubts concerning deprescribing and expressed confidence in their general practitioner. Our findings indicate a fundamental commitment to deprescribing LTX. For an adherent process in general practitioners’ practices, a strategy that considers patients’ worries and concerns seems feasible.

## KEY MESSAGE

Patients with subclinical hypothyroidism are open to having levothyroxine deprescribed if they are advised to do so by their general practitioner.

Targeted medical information, a stepwise approach, and close supervision by their general practitioner were identified as enablers.

## Introduction

Levothyroxine (LTX) is one of the most prescribed medications worldwide [1]. The primary purpose of LTX is to treat hypothyroidism, both manifest and occasionally subclinical. The diagnosis of subclinical hypothyroidism (SH) is based on an aberration of laboratory findings and is defined by an elevated serum-level of the thyroid stimulating hormone (TSH) while the free thyroxine (FT4) level is within normal range [2,3]. SH infrequently (2–5% annually) develops into manifest hypothyroidism [4]. International studies show an SH prevalence of 0.4–16.9% in the population, with variations depending on sex and age as well as geographic region [1,3,5]. The necessity of medical treatment and the indications to treat SH are the subject of historical changes and controversial debates [3,6].

Current clinical guidelines, both in Germany and internationally, suggest that patients with TSH levels between 4.0 and 10.0 mIU/L should not be routinely offered treatment with LTX, especially in the absence of clinical symptoms [7,8].

Studies have shown that the use of LTX has no beneficial effect on mortality, morbidity, or quality of life in patients with SH [9]. Despite this and the significant medical side effects associated with the use of LTX (e.g. cardiac arrhythmias, osteoporosis, fractures, iatrogenic hyperthyroidism [10–12]), the number of prescriptions is increasing worldwide [1]. A considerable proportion of LTX prescriptions in general practitioners’ (GPs’) practices in Germany are based on long-ago, now unclear indications that often result in long-term use of LTX – even in patients with SH [13]. Consequently, LTX contributes to the widespread phenomenon of polypharmacy, especially in the elderly population [2].

In Germany, LTX remains one of the most frequently prescribed drugs [13]. Current data suggests that almost 5 million people in Germany (population of 83 million) were treated with LTX in 2022, corresponding to more than 1458 million daily doses of LTX alone, indicating massive overtreatment [14]. The need to reduce LTX prescriptions is thus obvious.

Deprescribing means “the process of withdrawal of an inappropriate medication, supervised by a health care professional with the goal of managing polypharmacy and improving outcomes” [15]. However, although a systematic review and meta-analysis by Burgos et al. suggested that deprescribing was feasible and successful in over a third of patients treated with LTX, the study also highlighted the absence of a “systematic process/framework” [16] for deprescribing LTX. To the best of our knowledge, there is to date no evidence-based standardised procedure for deprescribing LTX in patients with SH in primary care. The DELTA PIA (Deprescribing of levothyroxine in subclinical hypothyroidism – a participatory intervention approach) research project aimed to close this gap. DELTA PIA was a collaborative project of University of Leipzig and the TUD Dresden University of Technology, both located in Germany. Following a participatory approach [17], the DELTA PIA project involved patients, GPs, and medical assistants (MAs). Involvement and consensual cooperation with the research subjects (i.e. patients) were the basic premises of our research process. Consequently, the first step in developing a deprescribing strategy was to understand the enablers and barriers of these groups, as there is little knowledge about patients’ and carers’ perspectives regarding the deprescribing of LTX in specific [13]. In a second step, a deprescribing strategy was planned based on these findings in a participatory approach with a second round of focus group discussions that enabled exchange and communication between patients and GPs. This paper specifically considers the patients’ perspective on motivations and barriers regarding the deprescribing of LTX. Both the results of GPs’ and MAs’ experiences and views, as well as the consented deprescribing strategy for LTX in primary care will be published separately.

This (first) part of the project aimed to answer the following questions:

1. What experiences with deprescribing LTX do patients with SH have?
2. Which enablers and motivations as well as obstacles and barriers are relevant for patients with SH in terms of deprescribing LTX?
3. What general conditions do patients with SH consider concerning the deprescribing of LTX?

## Methods

### Study design

To obtain in-depth perspectives of enablers and barriers of patients with SH to deprescribing of LTX, we conducted two structured focus group discussions (FGD) with patients with SH or experiences with SH and/or deprescribing as well as two mixed FGD with both, patients and GPs in April and May 2024. Patients who participated in the first round of focus groups were encouraged to return for the mixed focus groups to approve and further develop the results. In the research process, we were guided by the Consolidated Criteria for Reporting Qualitative Research (COREQ) checklist [18].

### Interview guide development

The guide for the semi-structured focus group discussions was self-developed by the interdisciplinary DELTA-PIA research team based on intensive literature review and team discussions. The guide for the patient group was aligned with the guide for the physician groups and vice versa. The interview guide was presented at a patient advisory board meeting at Leipzig University and reviewed by the participants for comprehensibility. All research group members approved the final version of the guide. The semi-structured guide contained 4 central items (Table 1), as well as instructions for the researchers and a proposed timetable.

**Table 1.** Semi-structured interview guide.

Introductory question: What are your experiences to date with thyroid medication (levothyroxine)?
Case vignette: Imagine that I am your GP. After we have discussed your routine appointment today and your currently good TSH value, I will now say the following: “You’ve been taking L-thyroxine for quite a while now. Your blood levels have been stable for a while, so I could imagine deprescribing your medication.”
Under what circumstances would you agree to deprescribing the medication, or what would be your arguments in favour of deprescribing?
What concerns would you have about deprescribing the medication?

### Recruitment and selection of study participants

Participants were recruited via GP practices within the local practice-based research networks in Leipzig (Research Practices Halle-Leipzig – RaPHaeL) and Dresden (Practice-based Research Network Dresden/Saxony- SaxoN). GPs and MAs of those practices recruited eligible patients via specific inclusion criteria (subclinical hypothyroidism AND absence of thyroid struma, thyroid carcinoma, and manifest hypothyroidism AND treatment with LTX OR previous treatment with LTX). Members of the patient advisory boards of the research networks were also invited to take part. The patient advisory boards were founded to support participatory research and to bring the patient perspective into research projects. The approach of integrating both affected individuals and trained patient representatives served the overarching goal of identifying common, coordinated and pragmatic positions as a basis for developing a deprescribing process in the later course of the project. Participants provided written consent to take part. For each focus group discussion participants received an expense allowance. An ethical review and approval were waived by the Ethics Committee of the Faculty of Medicine Carl Gustav Carus at Technical University Dresden on January 19, 2024, due to the characteristics of this study.

### Sample description

A total of 13 patient participants were included in the study (first round: FGD1, patients *n* = 5; FGD2, patients *n* = 7; second round: FGD3, patients *n* = 4, GPs *n* = 3; FGD4, patients *n* = 6, GPs *n* = 4). Most, but not all patient participants, returned for the second round, and one new patient was also recruited. The majority of participants were female and living in large cities with > 100.000 residents. Five of the participants were members of the local patient advisory boards. Most, but not all participants were currently taking LTX. Those patients are indicated in citation with “L”. Three participants had already tried to deprescribe LTX, either on their own or based on a recommendation from their GPs. In contrast, three participants did not have any experience with SH or LTX. Instead, they reported personal experiences with other long-term medications and/or with deprescribing. Those patients are indicated in citation with “I”. In Table 2, a detailed description of the sample is given.

**Table 2.** Sample description.

Patient characteristics (*n* = 13)	Value
Age in years, *M*±*SD*	56.0 ± 20.8
Gender	
Female, *n*	9
Male, *n*	3
Diverse, *n*	1
Residence location	
Large city^1^, *n*	8
Medium city^1^, *n*	4
Small city^1^, *n*	0
Rural^1^, *n*	1
LTX intake	
Previous	1
Ongoing	9
Deprescribing experience	
LTX	3
Other medication	1
GP characteristics (*n* = 7)	Value
Age in years, *Median (Min; Max)*	39.0 (28;53)
Gender	
Female, *n*	5
Male, *n*	2
Diverse, *n*	0
Workplace location	
Large city^1^, *n*	3
Medium city^1^, *n*	1
Small city^1^, *n*	1
Rural^1^, *n*	2
Status	
GP specialist	6
GP resident	1
Type of employment	
Self-employed	2
Employee	5

^1^ Large city (> 100.000 residents); Medium cities (>20.000–100.000 residents); Small city (5.000–20.000 residents); Rural (< 5.000 residents).

GPs who took part in the mixed group discussions were not involved in the treatment of the patient participants. Their sociodemographic data can also be found in Table 2.

### Focus group discussions

The focus group discussions lasted approximately two hours. The discussions were conducted locally at both research institutions in person and digitally *via* the video conference tool Zoom (Zoom communications, Inc., San José, California, USA). The participants were encouraged to engage in discussion among themselves and share their perceptions with minimal instructions by the attending researchers in both local places.

The focused outcomes of the study were enablers as well as barriers of patients with SH regarding a potential or already experienced deprescribing of LTX. The participants assigned points to the responses to items 3 and 4 based on their significance and relevance, thus establishing a priority for these responses. Afterwards, the results were discussed and consensus was reached.

We aimed to collect data from the patients themselves while also fostering reciprocal communication with GPs in mixed focus groups. This facilitated communication among the patients themselves, along with mutual inspiration from the viewpoints of GPs. In a second round of FGDs, outcomes were therefore presented, discussed, and agreed upon in mixed group discussions with GPs. The participants provided feedback to the summarised results and their agreement with the findings. The main focus of these groups was in reconciling ideas and developing a deprescribing strategy, which will be published elsewhere.

The focus group discussions took place without any interruptions or problems. They were recorded, transcribed and pseudonymised by the participating researchers. All transcripts and audio data were stored on a secure server and only accessible for analysis by the researchers. The transcription took place externally via the AI-based transcription tool *Transkripto.de* (Amberscript Global B.V., Amsterdam, Netherlands) and was randomly checked for correctness and plausibility by members of the research team.

### Analysis

The data were analysed by a member of the research team (MR), following the method of qualitative content analysis by Kuckartz [19]. The coding tree was established and clustered using MAXQDA^®^ (Verbi Software, Berlin, Germany) based on the FGD protocols and whiteboard documentations, taking into account the patients’ prioritisations. The structure of the coding tree corresponded to the research questions introduced before and was therefore divided into experiences with LTX or deprescribing, enablers and barriers of deprescribing, as well as the perceived conditions for a future withdrawal. The coding tree was finalised in a cross-check by another member of the research team (SH) and approved by the whole research team. The consented coding-tree was then applied to all FGD transcripts, which did not lead to any further modifications.

## Results

The results are based on the research aims and the analytical arrangement of the coding tree as well as the prioritisation in the focus group discussions.

### Individual experiences with LTX and deprescribing

The majority of participating patients were taking LTX for several years without being able to recall a clear initial indication. However, a small number of the patients who took part in the study had already discontinued LTX or at least attempted to discontinue. The deprescribing procedure either was conducted successfully or was interrupted by the patients due to alleged symptoms.

Basically, the patients stated that they had little or no knowledge about hypothyroidism and their LTX medication, including possible side effects.

‘Honestly, I don’t know anything about the side effects, I’ve never been told. I’ve never asked what are or could be side effects? I didn’t ask my doctor, because I’ve been taking it for so long. It’s like a habit, like brushing my teeth, taking this pill in the morning without thinking about it.’ (P2, female, L)

The participants explicitly stated that their long-term use of LTX had led them to adjust their daily routines to the medication and its intake schedule. For example, they described planning their daily LTX intake, managing their pills before going on holiday, or scheduling follow-up appointments at the GP practice for prescription renewals. Many of the participants described this health management as a burden.

### Enablers of deprescribing LTX

Most participating patients expressed their willingness to deprescribe LTX and expressed their enablers for doing so. For most, a recommendation from their GP would be the primary motivation for deprescribing LTX. Trust in the advice of the GP was a recurring topic in the focus group discussions.

‘Because the general practitioner knows you well. He also knows all of my other problems, over a longer period of time. […] I would follow his recommendations. […] It really is a question of trust.’ (P6, female, L)

By deprescribing LTX, patients mentioned the advantage of reducing the overall number of medications taken, including possible side effects. They viewed deprescribing of LTX as a positive step towards supporting their own health and as a way to reduce the burden of medication-related ‘chemicals’ in the body.

‘Every drug has a side effect, if I take one less, I have one less side effect or potentially ten. […] Every drug that you can take less of and the body functions is potentially less chemistry in the body.’ *(P3, male, L)*

Additionally, patients emphasised the benefit of deprescribing to improve their quality of life. As an enabler, patients highlighted the advantage of time and money savings.

Participants often described the reduction in medication as a form of *‘freedom’* (P9, female, I) in terms of being free from the organisational burdens caused by daily medication intake.

In conclusion, it can be stated that the patients considered deprescribing as an advantage for both their personal lives and the alleviation of stress associated with individual health management.

### Barriers of deprescribing LTX

Patients considered certain obstacles regarding deprescribing of LTX. Generally, the participants expressed distrust towards deprescribing, particularly against the background of their long-time usage of LTX. Patients noted that a sudden recommendation of change in their medication could affect their confidence in their GP. One patient mentioned, the advice for deprescribing by the GP

‘could lead to a loss of trust in the doctor. It [the medication] was prescribed and now it is being questioned, whether that has a negative effect on trust, I honestly don’t know […].’ (P3, male, L).

Besides this general uncertainty, most of the participants mentioned their concerns about assumed symptoms that may occur after deprescribing LTX. The assumed symptoms were associated with general symptoms of overt hypothyroidism [20].

‘Someone has convinced you that the weight gain is related to the thyroid gland. There is a fear; it’s in your subconscious and in your head.’ (P5, female, L)

The concern was not based on the patients’ actual knowledge of their disease, but rather on general assumptions.

Patients also feared that they would not receive sufficient support and information from their GP during the deprescribing process. The prevailing concern was that their own safety was at risk. In relation to potential (re-)appearing symptoms, patients were worried that those symptoms could remain unnoticed due to a lack of information and supervision on the part of the GPs. Additionally, patients also feared inconsistent recommendations from different doctors. Most of the patients received additional treatment from an endocrinologist or other health practitioners (e.g. alternative practitioners or nutritionists). Disagreement among doctors could lead to patients′ distrust and uncertainties regarding their own decisions:

‘It’s the reasoning, why does the GP say it this way and why does the specialist say it differently? If it’s the opposite, that would be dramatic, perhaps only nuances are different. The responsibility lies with you to say I trust this or that. I think that would be a difficult decision.’ (P2, female, L)

Generally, patients were sceptical and had concerns about sudden changes in their everyday life including changes in regular medications. Most of the patients experienced a long-term treatment with LTX. A change of common structures of behaviour was emphasised as “uncertainty”.

In conclusion, deprescribing meant a sudden change in medication and therefore an unpredictable change in patients’ lives. This led to uncertainties, mistrust, and could risk the process of an adherent deprescribing.

### General conditions of deprescribing

Patients regarded their own consent as a primary condition of deprescribing. If they did not feel comfortable during the process, they would consider the advantages of deprescribing LTX as less important. The patients emphasised that a shared decision making for and an agreed upon procedure of discontinuation between them and their GP was essential. In the case of co-treatment by a specialist (usually endocrinologist, and in some cases other consulted stakeholders, e.g. alternative practitioners), ideally, the recommendation for deprescribing should be confirmed by the co-treating specialist. In case of disagreement, some participants expected to have the option of a third-party opinion. However, most patients were willing to follow their GP’s advice first and foremost, as they trusted him or her the most.

‘It’s a question of who you see more often. The GP knows you better than the specialist you see once a year.’ (P3, male, L)

Fewer participants would prefer to follow the advice of the specialists (especially endocrinologists) or make an individual decision.

‘I […] say the doctor can give advice, but in the end, I have to decide for myself whether I do it or not.’ (P9, female, I)

To deprescribe successfully, the participants highlighted a trustful relationship with their GPs. They emphasised a long-term relationship and a consistent recommendation as being necessary. Other important parameters of trustfulness were considered as an adequate framing of deprescribing: this included education about SH and LTX, information on side effects, as well as advantages and disadvantages. Patients preferred to receive written information via flyers from their GP.

Patients preferred a gradual and closely managed deprescribing process. Immediate discontinuation was out of the question for most, as it triggered fears of uncontrollable side effects. A stepwise approach was expected to be regularly monitored by the GP. The exclusion of comorbidities played a decisive role for patients, too. Patients considered the assessment of comorbidities and TSH levels, along with a trusting relationship with their GP, to be essential components of a safe and well-managed deprescribing process.

‘I would be interested in it not being a shot in the dark, but that you have the feeling that if I put it down, I have x time units until the next review date.’ (P6, female, L)

The possibility of stopping the deprescribing process was a basic premise.

## Discussion

### Main findings

Current research indicates a significant overtreatment with LTX in patients with SH. The results of this study show that most patients had not questioned their long-term use of LTX and were therefore sceptical about a possible deprescribing. On the other hand, patients emphasised their general motivation and willingness to discontinue LTX. The potential benefits of deprescribing a medication outweighed the concerns arising from the discontinuation process.

The benefits of deprescribing were the main focus for patients. Financial and organisational relief, as well as the reduction of possible side effects of long-term medication would be the most important reasons for them to start a LTX withdrawal procedure. Nevertheless, there were concerns and fears among patients that could obstruct the process of deprescribing. On the one hand, patients’ concerns were due to having relied on their long-term LTX treatment based on their doctor’s advice for many years. A sudden change in recommendations regarding their long-term medication could overwhelm them. Patients’ own knowledge also played a decisive role. Their knowledge about hypothyroidism often did not match current medical standards and guideline recommendations. For patients, therefore, a successful and adherent deprescribing required sufficient information about their condition, the indication for LTX treatment, and the process of deprescribing. The results show that adequate information could help to alleviate the uncertainties and fears of affected patients. The process of deprescribing in patients with SH does not need to be outsourced to medical specialists (e.g. endocrinologists). Most patients felt more comfortable in a trustful setting with their GP.

Our results are in line with previous research. Viniol et al. showed in 2013, that the indication for LTX after many years of treatment is often unclear, and that patients often take LTX permanently without questioning the lifelong treatment [13]. However, evidence on the indication, process, and success of deprescribing thyroid hormones remains scarce [16]. Deprescribing, on the other hand, is a major issue in current research, with more than 2800 entries retrieved by PubMed with the search term “deprescribing” alone.

Our results are consistent with current research on deprescribing in general. The important role of patients’ cooperation and positive attitudes for the success of deprescribing is well established [21–23]. Many studies have shown that patients are mostly open to having their medication load reduced, though to varying degrees [24–28]. Great differences could be observed between countries [25,26], but it seems otherwise difficult to identify patient groups who are specifically interested in stopping their medication due to inconsistent findings [26–28].

The systematic review of Reeve et al. showed [22] that the process of cessation itself is a key factor in obtaining patients’ approval. Lack of time, information, and support were identified as major barriers reported by the patients. Additionally, as observed in our study, concerns about side effects or the recurrence of symptoms caused by the condition the medication was intended to treat were noted. Fear of side effects of the medication to be discontinued, costs, and associated inconveniences were named as enablers for deprescribing. As in our study, the authors highlighted the importance of a trustful relationship with the caregiver as a major condition. The role of communication and trust to the GP has been highlighted in other reviews as well [29,30]. Though clear protocols on how to proceed in detail are still lacking, ideally tailored for patient and medication groups, and based on clinical trials [21,31], there is broad consensus that patient-centred approaches will be most promising [21,32]. Consequently, understanding patients’ views, attitudes, barriers, enablers for deprescribing LTX is an important step in the process of designing, testing, and implementing protocols for an evidence-based approach to deprescribing LTX in patients with SH.

### Strengths and limitations

To the best of our knowledge, this study examines the hitherto little researched topic of patients’ perspectives on deprescribing LTX in GP practices in Germany. The strength of the study is the intensive involvement of patients in the study as well as the reciprocal process of discussing, inspiring, and deciding in collaboration with GPs.

In terms of study limitations, the recruitment procedure was partially challenging. Due to difficulties in finding patients with SH, it was necessary to extend the sample of patients to hypothyroidism in general or patients with experiences in deprescribing. Our sample of 13 patients included three patients that comply with these extended characteristics. Three participating patients were members of the local patient advisory boards and were affected by chronic diseases not related to the thyroid gland. The analysis results do not show any differences compared to the 10 patients with SH. That implies an empathic understanding of barriers, obstacles, enablers, and motivations of deprescribing a long-term medication. Second, we included more patients from larger cities, mainly due to both participating universities being located in metropolitan areas. Nonetheless, the population in Germany is highly urbanised, with 77.9% of the population living in urban areas [33]. Similarly, we included more women in our study, which could partly be due to more females being affected from SH [34]. Medication count and information on other medical conditions was not documented in our study. We suggest that further research should focus both men and people living in rural areas and consider chronic medical conditions.

While there may be critical voices who view the deliberate inclusion of a smaller subset of patients without the specific condition but with experience participating in patient advisory boards, as well as GPs, as limiting, we believe that expanding the groups to include additional perspectives was enriching.

Furthermore, mixed groups of patients as well as GPs carry the risk of social desirability bias. To best control for group dynamics and imbalances, we used semi-structured methods as well as moderation by experienced researchers. Though based on an interview guide, this included the opportunity to deepen the topics and ask additional questions for better approaching the truth.

### Implications

Our study showed that patients are generally positive and open towards deprescribing LTX. Emphasising the benefits for patients’ quality of life could be a starting point for the doctor–patient consultation. Individual patient caveats should be considered and the recommended procedure adapted to the individual patients’ conditions. Based on the findings about barriers, enablers and motivations as well as discussed general conditions of deprescribing, the project team is currently creating a strategy for deprescribing LTX, which is agreed upon by patients as well as GPs. A clinical trial to assess the effectiveness of the deprescribing strategy in patients with SH is planned.

## Conclusion

Patients recognise potential benefits of deprescribing, and assume that they could improve their quality of life by discontinuing the treatment with LTX. A trustful relationship with their GP and sufficient communication about the benefits and risks of their condition and the deprescribing process are seen as the basic premises. Patients prefer that deprescribing happens gradually and that laboratory values are checked regularly. Deprescribing should be a process of shared decision-making.

## Data Availability

The data that support the findings of this study are available from the corresponding author upon reasonable request.
